# Integrated Design of Spindle Speed Modulation and Cutting Vibration Suppression Controls Using Disturbance Observer for Thread Milling

**DOI:** 10.3390/ma14216656

**Published:** 2021-11-04

**Authors:** Syh-Shiuh Yeh, Chai-Wei Chen

**Affiliations:** 1 Department of Mechanical Engineering, National Taipei University of Technology, Taipei 10608, Taiwan; 2 Institute of Mechatronic Engineering, National Taipei University of Technology, Taipei 10608, Taiwan; allenchen0112@gmail.com

**Keywords:** spindle speed modulation, cutting vibration suppression, disturbance observer, thread milling

## Abstract

In thread milling, there exists a trade-off between thread manufacturing efficiency and thread quality. In this study, an integrated design of spindle speed modulation (SSM) and cutting vibration suppression (CVS) controls using a disturbance observer were developed to simultaneously ensure superior quality and high manufacturing efficiency. The proposed integrated design not only controls the cutting torque while suppressing cutting vibrations but also ensures cost-effectiveness and mitigates the installation problems prevalent in existing sensor-based methods. The SSM control uses a disturbance observer to estimate the cutting torque required on the spindle during thread milling. The estimated cutting torque is used as a feedback signal so that the SSM control can modulate the spindle speed to make the cutting torque achieve a preset torque command. To further avoid cutting vibrations in thread milling, the CVS control analyzes the estimated cutting torque, detects the occurrence of cutting vibrations, and then adjusts the torque command of the SSM control to suppress the cutting vibrations. In this study, thread milling experiments were performed on a computer numerical control milling machine using the workpiece with stacked materials. The feasibility and performance of the proposed integrated design were validated by experiments.

## 1. Introduction

The thread manufacturing method of thread milling has been in practice for a long time and is used to manufacture high-strength and high-accuracy threads with good surface characteristics [1,2,3,4]. However, factors influencing the manufacturing process, such as cutting force, cutting torque, and cutting stability, should be further investigated to ensure high thread manufacturing efficiency and superior thread quality. 

The cutting force, cutting torque, and cutting stability of milling have been extensively studied in past decades; however, there were only a few studies on thread milling. The existing methods for milling use different sensors to achieve precise cutting force control, accurate cutting torque control, and efficient cutting vibration suppression. However, the sensor-based methods have problems with installation and high implementation costs. Even though some sensorless methods have been developed to avoid the aforementioned problems, cutting force control, cutting torque control, and cutting vibration suppression were studied individually and independently. Unlike the existing methods, the method proposed in this study has the following distinguishing features:

- A disturbance observer (DOB) is used to simultaneously estimate the cutting torque and detect cutting vibrations.
- An integrated design is used that combines spindle speed modulation (SSM) and cutting vibration suppression (CVS) controls to control the cutting torque while avoiding cutting vibrations by adjusting the spindle speed.
- The integrated design ensures high thread manufacturing efficiency and superior thread quality without using additional sensors and thereby avoids the problems present in sensor-based methods.

Moreover, this study systematically details the integrated design while considering the dynamics of the spindle motor and disturbance observer. A computer numerical control (CNC) milling machine was used to perform the thread milling experiments using workpieces with stacked materials and the experimental results were used to validate the feasibility and performance of the proposed integrated design.

The remainder of this paper is organized as follows. Section 2 presents the research related to this study. Section 3 presents the integrated design developed in this study, including the SSM control design and CVS control design. Section 4 presents the experimental results used to validate the feasibility and performance of the integrated design. Section 4 also describes the experimental setup used in this study and presents the results of the thread milling experiments on workpieces with stacked materials. Section 5 presents the conclusions of this study.

## 2. Related Works

The cutting force and cutting torque have a significant influence on thread milling efficiency and thread quality. Therefore, some studies have focused on the effects of cutting force and cutting torque dynamics during thread milling. For example, Araujo and Fromentin [5] studied the relationship between the cutting forces and tool deflection in a typical internal thread machining and proposed a tool trajectory planning method for the hard-to-cut materials by considering the tool deflection characteristics. Sharma et al. [6] studied the influence of tool geometry on the cutting forces and concluded that the cutting forces can be minimized by applying a proper penetration strategy for the cutting tool. Araujo et al. [7] investigated the influence of thread geometry, cutting conditions, and tool angles on the cutting force and cutting torque and reported that their results can be used for tool optimization and to determine the thread milling parameters. Lee et al. [8] created a cutting force model of thread milling and applied it to the cutting parameters to increase the productivity of thread milling. Fromentin et al. [9] studied the influence of mill penetration during thread milling on the accuracy of the threads produced. They presented appropriate thread milling penetration strategies to manufacture accurate threads. Neshta et al. [10] constructed a multipoint-cutting noncore tool and developed an algorithm to calculate the trajectory parameters and efficiently machine internal rope threads. They further developed a procedure to compensate for the wear of the non-core tool to improve the dimensional accuracy of the rope thread profile. To improve surface roughness, Doudkin et al. [11] adopted an experimental design method to determine the optimal cutting parameters for double threading with the workpiece material of Grade 5 titanium alloy. They further obtained a regression equation to correlate the surface roughness and thread cutting parameters. Therefore, the thread milling efficiency and thread quality in thread milling can be improved by designing appropriate cutting tool path and trajectory, tool geometry and penetration strategy, and thread milling conditions such as spindle speed, cutting feed, and axial depth of cut. However, it is difficult to modify these designs and adjust them in real-time owing to the diverse machining characteristics in thread milling and variations in the workpiece materials, cutting tool materials, and machining environment. Therefore, the development of cutting force and cutting torque controls that can adjust thread milling conditions in real-time is essential for further improvement of thread milling efficiency and thread quality in thread manufacturing.

The applications of cutting force and cutting torque controls in milling have been studied for a long time. For example, to achieve constant cutting force, Maneerat et al. [12] developed an adaptive cutting force control that senses the cutting force during milling and controls the feed rate of a CNC milling machine. Davis et al. [13] developed an adaptive robust control for peripheral milling to reduce contour error while regulating the cutting force in the presence of model error and external disturbances. Denkena and Flöter [14] integrated the models of feed drive dynamics and cutting process to develop a proportional-integral-derivative-based adaptive control that regulates the cutting force in a milling machine. Zuperl et al. [15] developed an on-line adaptive control that adjusts the feedrate override percentage of a CNC controller to achieve a high metal removal rate and maintain the cutting force to a given reference value. Using the current signals obtained from the servo-amplifiers of feed drive axes and the frequency inverter of the spindle, Gómez-Loenzo et al. [16] developed a sensorless method to estimate the cutting force, control the feedrate overrate of the retrofitted milling machine, and reduce the cycle time. Kim and Jeon [17] developed a fuzzy-logic control, through analysis of the motor currents of the feed drive axes and spindle in a vertical machining center, to increase the metal removal rate while maintaining a constant cutting force using the cutting force estimated by the spindle motor current. Cutting force control with a dynamometer to measure the actual cutting force in milling exhibits superior control performance [12,15,18,19]. However, owing to the high implementation cost and interference problem during the dynamometer installation, milling machines seldom use a cutting force control with a dynamometer. Therefore, to overcome the aforementioned problems pertaining to the use of dynamometer, cutting force and cutting torque controls were developed in previous studies with reference to force models and current signals. Based on the cutting mechanics and tool geometry, several cutting force models were developed and adopted in the design of cutting force control [13,14,20,21]. However, owing to the diverse machining conditions and characteristics in actual milling, it is difficult to realize the model-based cutting force control for general milling processes. To address the problems associated with the use of dynamometer and force models, cutting force and cutting torque controls using current signals obtained from feed drive and spindle motors were developed [16,17,22,23]. However, some factors, such as friction and dynamics of the feed drive and spindle motors, could contaminate current signals and thus degrade the cutting force and cutting torque control performance. Therefore, as the cutting force and disturbance are related, a disturbance observer was adopted to estimate the cutting force in milling [24,25] and several studies have focused on the applications of chatter detection [26,27], machining type detection [28], cutting force monitoring [29], and tool fracture detection [30]. In this study, in contrast to previous studies, a feedback control method integrated with a DOB was developed to estimate and feedback cutting torque, modulate spindle speed, and make the cutting torque in thread milling achieve a preset torque command. The proposed method also considers the dynamics of the spindle and DOB to achieve precise cutting torque control.

Although precise cutting force and cutting torque controls in thread milling can improve milling efficiency and thread accuracy, cutting vibrations can deteriorate the thread surface. Therefore, Wan and Altintas [31] analyzed the mechanics and dynamics of thread milling and obtained a correlation between the thread milling stability and spindle speed, axial depth of cut, cutter path, and tool geometry. As cutting vibrations have a significant influence on milling efficiency and thread quality, many issues pertaining to cutting vibrations, such as mechanics, dynamics, detection, identification, control, and suppression, have been extensively studied in recent decades. For example, Han et al. [32] employed rotational invariance techniques to extract the frequency characteristics of acceleration signals and designed a fuzzy control algorithm to adjust the spindle speed of the machine tool and thereby suppress cutting vibrations. Liu et al. [33] integrated the empirical mode decomposition and wavelet packets decomposition to address the problem of modal aliasing in chatter detection; in the method adopted by them, the intrinsic mode functions corresponding to the chatter frequencies were selected and used to reconstruct signals, which were further processed for feature extraction. For on-line chatter recognition and suppression, Jin et al. [34] integrated the rotational invariance technique and hidden Markov model to develop a chatter recognition method and subsequently developed a fuzzy control method to adjust the spindle speed of the machine tool and suppress the chatter in milling. Han et al. [35] analyzed the triaxial cutting forces and acceleration signals during milling and used a dynamic model of the milling system to establish a relationship between the chatter frequency and spindle speed. In their study, they referred to the fast Fourier transform (FFT) result of the sensing signals; they examined and modified the cutting parameters, on the basis of the result, to suppress the milling chatter. Santos et al. [36] considered the activation of spindle electronic control to improve the accuracy of the stability lobe diagram (SLD). The SLD was used to select the appropriate spindle speed and depth of cut to avoid any chatter phenomenon during milling. Chen et al. [37] used an accelerometer to obtain the vibration signals during milling and designed a fuzzy control scheme to adjust the spindle speed to effectively suppress chatter and improve the workpiece surface texture. Merdol and Altintas [38] created a virtual environment for milling by computer-aided manufacturing to determine the allowable depth and width of a cut to avoid chatter; the post-processing system that they developed could adjust the feedrate and spindle speed and thereby increase the productivity of the manufactured parts. Recently, Yue et al. [39] summarized the current developments in chatter prediction, identification, and suppression in milling processes; moreover, the effects of process damping, tool runout, and gyroscopic effect on milling SLDs were analyzed for the improvement of chatter prediction. Yue et al. [39] also summarized the methods for the detection of cutting vibrations in milling using different sensing signals such as acceleration, cutting force, acoustic emission, and vision; furthermore, they discussed the problems of using sensing signals, such as the implementation cost and selection of installation positions. Therefore, the DOB was used to detect the cutting vibrations in milling. Yamato et al. [26] developed an adaptive method that integrated a DOB to monitor the chatter in a parallel end-milling process to adjust spindle speed for chatter suppression in real-time. In their method, the DOB was used to estimate the cutting force and the sliding discrete Fourier transform processed the estimated cutting force for monitoring the chatter and obtaining the chatter frequency. Based on the servo information obtained from the spindle system, Kakinuma et al. [27] used a DOB to estimate the disturbance torque exerted on the spindle motor; the estimated disturbance torque was used to detect the chatter in the end-milling process and to identify the self-excited and forced vibrations using different filters. Therefore, in this study, to address the problems of using sensor-based methods for the detection of cutting vibration, a DOB was used to detect the cutting vibrations. Moreover, in contrast to current studies, this study integrated the cutting torque control and cutting vibration detection using a DOB to achieve precise cutting torque control while avoiding cutting vibrations. 

In this study, an integrated design of SSM and CVS controls with a DOB for thread milling was developed. Using the proposed integrated design, the spindle motion characteristics can be modulated for improving the thread manufacturing efficiency and thread quality. The DOB was used to estimate the cutting torque and the estimated cutting torque was used as a feedback signal to design the SSM control for modulating the spindle speed. The estimated cutting torque was subtracted from the preset torque command to generate the torque error signal and the SSM feedback controller used the torque error signal to calculate the amount of modulation required to ensure that the cutting torque during thread milling was maintained at the preset torque command. Setting a low preset torque command ensures a good thread surface; however, the thread manufacturing efficiency becomes low owing to the prolonged milling time. Although setting a high preset torque command can shorten the thread milling time, the milling process can induce cutting vibrations and deteriorate the thread surface. Therefore, based on the SSM control structure, the CVS control was developed to mitigate the influence of cutting vibrations on the thread surface. The CVS control used FFT to analyze the estimated cutting torque signal and determine the occurrence and frequency of the cutting vibrations. When cutting vibrations occurred, the CVS control adjusted the preset torque command and suppressed the cutting vibrations. The thread milling experiments were performed on a CNC milling machine to validate the performance of the integrated design. The DOB experiment was performed to validate the accuracy of the estimated cutting torque. The average value and root mean square value of the steady-state error in the cutting torque were less than 5% of the actual cutting torque. A workpiece with stacked materials was used to validate the feasibility of the SSM control. The SSM control was performed on the workpiece with different materials (AL6061 and AL7075) and the average value of the steady-state error in the cutting torque was less than 2%. The CVS control experiment was performed to evaluate the cutting vibration suppression effect during thread milling. The results showed that the manufactured thread surfaces on the workpiece with different materials did not exhibit apparent cutting marks. Thus, the thread milling experiments validated the feasibility and performance of the integrated SSM and CVS control designs. 

## 3. Approach

### 3.1. Spindle Speed Modulation (SSM) Control

**Figure 1:**
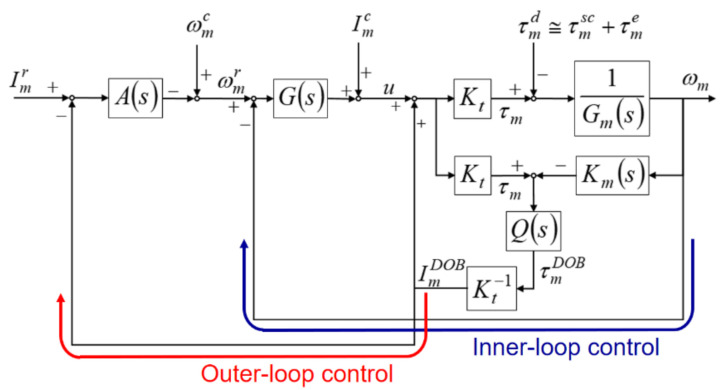
Structure of the spindle speed modulation (SSM) control.

Figure 1 shows the schematic of the SSM control structure designed in this study. When the spindle performs thread milling, the SSM control uses the DOB to estimate the cutting torque. Subsequently, the feedback controller modulates the spindle speed to ensure that the cutting torque estimated by the DOB is maintained at the preset torque command. Thus, the SSM control designed in this study has two parts: spindle speed control (inner-loop control) and cutting torque control (outer-loop control). 

The DOB was mainly used for the estimation of the cutting torque. In the DOB design, the following equations were used: $G_{m}\left(s\right)=J_{m}s+B_{m}$, where $G_{m}^{-1}\left(s\right)$ represents the transfer function between the net driving torque $\tau_{m}^{a}$ of the spindle and the actual speed $\omega_{m}$; $K_{m}\left(s\right)={\hat{J}}_{m}s+{\hat{B}}_{m}$, where $K_{m}\left(s\right)$ represents the estimated inverse transfer function between the net driving torque $\tau_{m}^{a}$ and the actual speed $\omega_{m}$. $Q\left(s\right)$ was designed as a first-order low-pass filter whose bandwidth was a ($Q\left(s\right)=\frac{a}{s+a}$). $K_{t}$ is the torque constant of the spindle motor and $\tau_{m}^{DOB}$ represents the cutting torque estimated by the DOB. The error between the estimated cutting torque $\tau_{m}^{DOB}$ and the actual cutting torque $\tau_{m}^{e}$ depends on the bandwidth of the low-pass filter $Q\left(s\right)$, the influence of disturbance torque $\tau_{m}^{sc}$, and the degree of approximation of $K_{m}\left(s\right)$ to $G_{m}\left(s\right)$. Here, $\tau_{m}^{d}$ denotes the external disturbance torque, which was mainly composed of the disturbance torque $\tau_{m}^{sc}$ and the actual cutting torque $\tau_{m}^{e}$.

The spindle speed control was used to control the speed of the spindle motor to ensure that the spindle maintained a good speed performance under the influence of external disturbance. The spindle speed control design was combined with the speed feedback proportional-integral (PI) control, DOB, and current compensation. The speed feedback PI control modulated the error between the spindle speed command $\omega_{m}^{r}$ and the actual speed $\omega_{m}$ with the $G\left(s\right)$ controller (PI controller) (i.e., Gs=Kvp1+1Tsi, where $K_{vp}$ is the speed feedback proportional gain and Ti is the integration time constant). The driving current of the spindle motor is the sum of the output of the $G\left(s\right)$ controller, current compensation, and output of the DOB. The effect of reducing the non-linear disturbance torque $\tau_{m}^{sc}$ synthesized by the Stribeck friction and Coulomb friction was limited because the DOB used in this study had a linear control structure. Therefore, the current compensation $I_{m}^{c}$ in this study reduced the influence of the nonlinear disturbance and increased the accuracy of cutting torque estimation.

The cutting torque control uses the estimated cutting torque as a feedback signal to ensure that the spindle motor can modulate the speed on the basis of the estimated cutting torque to maintain the cutting torque at the preset torque command. A torque-equivalent current method was used to design the cutting torque control. In the SSM control structure, as shown in Figure 1, $I_{m}^{r}$ is the equivalent current for the preset torque command and $I_{m}^{DOB}$ is the equivalent current for the estimated cutting torque $\tau_{m}^{DOB}$. In the cutting torque control design, the estimated current $I_{m}^{DOB}$ is subtracted from the current command $I_{m}^{r}$ to generate the current error. The cutting torque feedback controller $A\left(s\right)$ generates signals to modulate the spindle speed command $\omega_{m}^{r}$. The spindle speed command $\omega_{m}^{r}$ is the modulated signal generated by subtracting the output of the feedback controller $A\left(s\right)$ from the speed command compensation $\omega_{m}^{c}$. Therefore, the actual speed $\omega_{m}$ of the spindle can be changed to reduce the current error and maintain the cutting torque at the preset torque command. 

### 3.2. Control Design

The speed $G\left(s\right)$ and cutting torque feedback controller $A\left(s\right)$ influence the motion characteristics of the spindle; therefore, the effect of the structure of SSM control on the spindle speed was studied. 

Figure 1 shows the relationship between the electro-mechanical signal and the transfer function expressed as Equation (1) and Equation (2), respectively.

(1) $$K_{t}I_{m}^{DOB}=Q\cdot \left(\tau_{m}-K_{m}\omega_{m}\right)$$

(2) $$\tau_{m}=K_{t}u+K_{t}I_{m}^{DOB}$$

Equation (2) is substituted in Equation (1) to obtain Equation (3).

(3) $$K_{t}\left(1-Q\right)I_{m}^{DOB}+QK_{m}\omega_{m}=K_{t}Qu$$

Because

(4) $$\tau_{m}=G_{m}\omega_{m}+\tau_{m}^{d}$$

Equation (5) can be deduced from Equations (2) and (4).

(5) $$-K_{t}I_{m}^{DOB}+G_{m}\omega_{m}=K_{t}u-\tau_{m}^{d}$$

Moreover, Equations (6) and (7) can be deduced from Equations (3) and (5).

(6) $$\left[\begin{array}{cc} K_{t} & -1\\ K_{t}Q & 0 \end{array}\right]\;\left[\begin{array}{c} u\\ \tau_{m}^{d} \end{array}\right]=\left[\begin{array}{cc} G_{m} & -K_{t}\\ QK_{m} & K_{t}\left(1-Q\right) \end{array}\right]\;\left[\begin{array}{c} \omega_{m}\\ I_{m}^{DOB} \end{array}\right]\;$$

(7) $$\left[\begin{array}{c} u\\ \tau_{m}^{d} \end{array}\right]=\left[\begin{array}{cc} K_{t}^{-1}K_{m} & Q^{-1}\left(1-Q\right)\\ -\left(G_{m}-K_{m}\right) & K_{t}Q^{-1} \end{array}\right]\;\left[\begin{array}{c} \omega_{m}\\ I_{m}^{DOB} \end{array}\right]$$

Equation (7) describes the relationship between the signal ${\left[\begin{array}{cc} u & \tau_{m}^{d} \end{array}\right]}^{T}$ and the response ${\left[\begin{array}{cc} \omega_{m} & I_{m}^{DOB} \end{array}\right]}^{T}$ of the spindle. 

Figure 1 also shows the relationship between the control signal and the transfer function expressed as Equation (8) and Equation (9), respectively.

(8) $$u=I_{m}^{c}+G\cdot \left(\omega_{m}^{r}-\omega_{m}\right)$$

(9) $$\omega_{m}^{r}=\omega_{m}^{c}-A\cdot \left(I_{m}^{r}-I_{m}^{DOB}\right)$$

The current compensation $I_{m}^{c}$ is designed as Equation (10) to reduce the adverse effect of the nonlinear disturbance torque $\tau_{m}^{sc}$ and viscous friction torque.

(10) $$I_{m}^{c}=K_{t}^{-1}\cdot \left({\hat{\tau }}_{m}^{sc}+{\hat{B}}_{m}\omega_{m}^{r}\right)$$

Equations (9) and (10) are substituted into Equation (8) to obtain Equation (11).

(11) $$\begin{array}{l} u=\left[\begin{array}{cc} -G & \left(K_{t}^{-1}{\hat{B}}_{m}+G\right)\;A \end{array}\right]\;\left[\begin{array}{c} \omega_{m}\\ I_{m}^{DOB} \end{array}\right]\\ \;+K_{t}^{-1}{\hat{\tau }}_{m}^{sc}\\ \;+\left(K_{t}^{-1}{\hat{B}}_{m}+G\right)\;\left(\omega_{m}^{c}-AI_{m}^{r}\right) \end{array}$$

Subsequently, Equation (11) can be rewritten as Equation (12).

(12) $$\begin{array}{l} \left[\begin{array}{c} u\\ \tau_{m}^{d} \end{array}\right]=\left[\begin{array}{cc} -G & \left(K_{t}^{-1}{\hat{B}}_{m}+G\right)\;A\\ 0 & 0 \end{array}\right]\;\left[\begin{array}{c} \omega_{m}\\ I_{m}^{DOB} \end{array}\right]\\ \;\;+\left[\begin{array}{c} K_{t}^{-1}{\hat{\tau }}_{m}^{sc}+\left(K_{t}^{-1}{\hat{B}}_{m}+G\right)\;\left(\omega_{m}^{c}-AI_{m}^{r}\right)\\ \tau_{m}^{d} \end{array}\right] \end{array}$$

Here, Equation (12) describes the relationship between the signal ${\left[\begin{array}{cc} u & \tau_{m}^{d} \end{array}\right]}^{T}$ and the response ${\left[\begin{array}{cc} \omega_{m} & I_{m}^{DOB} \end{array}\right]}^{T}$. 

After the detailed derivation shown in the Appendix A, Equation (13) can be deduced from Equations (7) and (12).

(13) $$\begin{array}{l} \left(K_{m}+K_{t}G\right)\;\omega_{m}\\ +\left[1-Q-\left({\hat{B}}_{m}+K_{t}G\right)\;AK_{t}^{-1}Q\right]\;\left(G_{m}-K_{m}\right)\;\omega_{m}\\ =\left({\hat{\tau }}_{m}^{sc}-\tau_{m}^{sc}\right)-\left(1-Q\right)\;\tau_{m}^{e}\\ +\left({\hat{B}}_{m}+K_{t}G\right)\;\left[\left(\omega_{m}^{\mathrm{ref}}-AI_{m}^{r}\right)-\left(AK_{t}^{-1}+{\left({\hat{B}}_{m}+K_{t}G\right)}^{-1}\right)\;Q\;\left({\hat{\tau }}_{m}^{sc}-\tau_{m}^{sc}\right)-AK_{t}^{-1}Q\;\left({\hat{\tau }}_{m}^{e}-\tau_{m}^{e}\right)\right] \end{array}$$

In the discussion on control design, it was assumed that the system identification results had a good degree of approximation (i.e., $K_{m}≅G_{m}$, ${\hat{\tau }}_{m}^{sc}≅\tau_{m}^{sc}$, and ${\hat{\tau }}_{m}^{e}≅\tau_{m}^{e}$). Thus, Equation (13) can be approximated as Equations (14) and (15).

(14) $$\left(K_{m}+K_{t}G\right)\omega_{m}≅-\left(1-Q\right)\tau_{m}^{e}+\left({\hat{B}}_{m}+K_{t}G\right)\left(\omega_{m}^{ref}-AI_{m}^{r}\right)$$

(15) $$\omega_{m}≅\frac{\left({\hat{B}}_{m}+K_{t}G\right)}{\left(K_{m}+K_{t}G\right)}\left(\omega_{m}^{ref}-AI_{m}^{r}-\frac{\left(1-Q\right)}{\left({\hat{B}}_{m}+K_{t}G\right)}\tau_{m}^{e}\right)$$

Given that $K_{m}\left(s\right)={\hat{J}}_{m}s+{\hat{B}}_{m}$ and Gs=Kvp1+1Tsi, the characteristic equation can be obtained as Equation (16).

(16) $${\hat{J}}_{m}T_{i}s^{2}+\left({\hat{B}}_{m}+K_{t}K_{vp}\right)T_{i}s+K_{t}K_{vp}=0$$

From Equation (16), the damping ratio ζ and natural undamped frequency $\omega_{n}$ are obtained. Subsequently, the speed feedback proportional gain $K_{vp}$ and the integration time constant Ti can be expressed as Equation (17) and Equation (18), respectively.

(17) $$K_{vp}=\frac{1}{K_{t}}\left(2\zeta \omega_{n}{\hat{J}}_{m}-{\hat{B}}_{m}\right)$$

(18) $$T_{i}=\frac{1}{{\hat{J}}_{m}\omega_{n}^{2}}\left(2\zeta \omega_{n}{\hat{J}}_{m}-{\hat{B}}_{m}\right)$$

A feedback controller $A\left(s\right)$ was designed as Equation (19) to avoid any significant changes in the transient response of the equivalent current $I_{m}^{r}$ to the actual speed $\omega_{m}$ of the spindle.

(19) $$A\left(s\right)=K_{\alpha }\frac{\alpha }{s+\alpha }$$

In Equation (19), bandwidth α is adjusted to ensure that the actual speed $\omega_{m}$ of the spindle has an appropriate transient response with a short rise time and without any overshoot. The gain $K_{\alpha }$ adjusts the steady-state response of the equivalent current $I_{m}^{r}$ to the actual speed $\omega_{m}$ of the spindle. Therefore, on the basis of the actual speed response of the spindle, the SSM control design is summarized as follows:

- Proportional gain $K_{vp}$ and integration time constant Ti of the feedback controller $G\left(s\right)$ were designed to ensure that the spindle speed had an appropriate damping ratio and a natural undamped frequency.
- Bandwidth a the low-pass filter $Q\left(s\right)$ was designed to ensure that the DOB could reduce the adverse effects of the external disturbance on the spindle speed.
- Bandwidth α and gain $K_{\alpha }$ of the feedback controller $A\left(s\right)$ were designed to ensure that the spindle had appropriate transient and steady-state responses to the SSM control.

### 3.3. Cutting Vibration Suppression (CVS) Control

**Figure 2:**
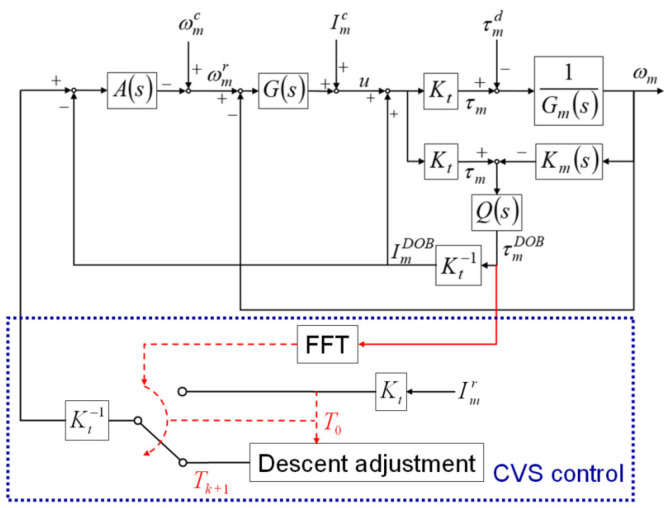
Cutting vibration suppression (CVS) control of the integrated design.

During thread milling, cutting vibrations can shorten the service life of the cutting tool and result in apparent cutting marks on the surface of the manufactured threads. Because the SSM control can maintain the cutting torque during thread milling at a preset torque command, cutting vibrations can be suppressed by properly scaling down the torque command. Therefore, in this study, the CVS control was developed to detect the occurrence of cutting vibrations and scale down the torque command of the SSM control to suppress the vibrations. The integrated design of the CVS and SSM controls is shown in Figure 2. The CVS control uses the FFT to analyze the cutting torque estimated by the DOB and detect the occurrence and frequency of the cutting vibrations. When cutting vibrations are detected, the CVS control scales down the torque command of the SSM control to suppress the cutting vibrations. 

In this study, the descent method with a constant step size, which is simple and has low computational time, was employed to adjust the value of the torque command of the SSM control, as shown in Equation (20).

(20) $$T_{k+1}=T_{k}-\gamma \cdot \Delta T,\;k=0,1,\ldots ,n,$$

where $T_{k+1}$ is the $\left(k+1\right)$th calculated value of the torque command; $T_{k}$ is the $\left(k\right)$th calculated value of the torque command; ΔT is the constant step; γ is the scaling factor and 0<γ<1; $T_{0}$ is the value of the torque command when the CVS control detects cutting vibrations. Multiple iterations of Equation (20) were computed until the CVS control did not detect any cutting vibration. The scaling factor γ has a significant influence on the response time of the CVS. A large scaling factor value results in a short response time of the CVS and low torque command. In contrast, a small scaling factor value can result in an appropriate torque command; however, the response time of the CVS is prolonged. Several factors, such as machining conditions, cutting tools, and workpiece materials, can influence the scaling factor. Therefore, before performing actual thread milling, several cutting tests are required to obtain an appropriate value of the scaling factor. 

## 4. Experiment

### 4.1. Experimental Setup

**Figure 3:**
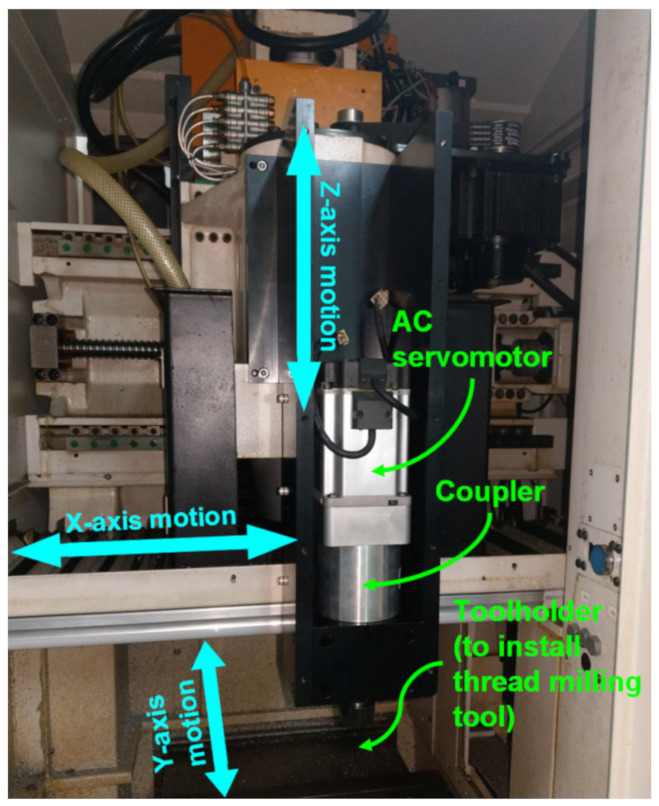
Computer numerical control (CNC) milling machine used in this study.

A CNC milling machine (Industrial Technology Research Institute, Taichung, Taiwan), mainly composed of a spindle and feed drive axes, was used for the thread milling experiments, as shown in Figure 3. The spindle and feed drive axes were equipped with AC servomotor packs controlled by an FPGA-based cRIO control console and input-output interface modules developed by National Instruments (NI). The control console, along with the NI LabVIEW software (LabVIEW 2014, National Instruments, Austin, TX, USA), provides operation functions such as a human–machine interface, signal measurement and feedback control, and recording and displaying the experimental data. Moreover, the control console uses the installed input-output interface modules, such as NI 9263 analog output module, NI 9401 digital I/O and encoder module, and NI 9215 analog input module, to send control commands to the AC servomotor packs and receive feedback signals with a sampling period of 1 ms. The control console sends control commands to the AC servomotor packs via the NI 9263 analog output module. Because a rotary encoder is directly coupled to the servomotor for sensing angular positions, the control console uses the NI 9401 digital I/O and encoder module to receive the encoder signals and decode the angular positions. The control console receives the analog sensing signals from the servomotor pack via the NI 9215 analog input module to obtain the actual speed and torque values of the servomotor. The feed drive axes comprise vertical (Z-axis) and planar axes (X-axis and Y-axis) so that they can move the thread milling tool (installed on the spindle through toolholder and coupler) in the machining space of the milling machine.

A Kistler 9129AA multicomponent dynamometer with a Kistler 5167A41 amplifier (Kistler, Winterthur, Switzerland) was used to measure the actual cutting forces generated during the thread milling experiments. A thread milling tool, with a length of 90 mm and shaft diameter of 12 mm, was adopted for thread milling cylindrical workpieces. The disk-type thread milling blade, with a 20-mm blade diameter and 60° blade angle, was made of tungsten steel (Yih Troun Enterprise Co., Ltd., New Taipei City, Taiwan). The workpiece materials were AL6061 and AL7075. Constant-speed experiment and moment of inertia estimation experiment [40] were conducted to estimate the friction parameters and the moment of inertia of the spindle, respectively. The experimental results are as follows: the viscous friction coefficient of the spindle was 0.4310 × 10^−3^ Nm/rad/s, the Coulomb friction coefficient was 0.0862 Nm, and the moment of inertia of the spindle (with the thread milling tool) was 1.4833 × 10^−4^ kg·m^2^.

### 4.2. Disturbance Observer (DOB) Tests

**Figure 4:**
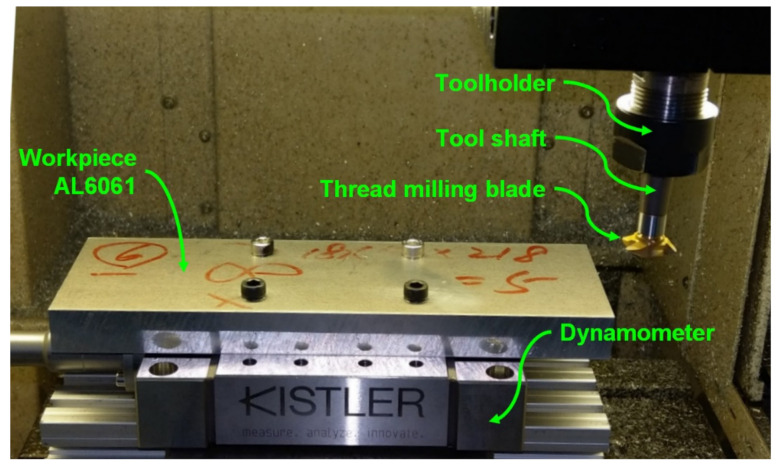
Experimental setup for the estimation of cutting torque using the disturbance observer (DOB).

Figure 4 shows the experimental setup of the DOB tests. The Kistler dynamometer was installed on the worktable of the milling machine and the test workpiece, aluminum alloy AL6061, was mounted on the dynamometer. During the tests, the thread milling tool performed the milling operation on the side of the test workpiece and the dynamometer measured the actual cutting forces during milling in order to obtain the actual cutting torque on the spindle. The cutting torque estimated by the DOB was compared with the actual cutting torque obtained by the dynamometer to validate the estimation performance of the DOB. In the tests, the cutting feedrate was 300 mm/min, the depth of cut was 1.5 mm, and the spindle speed was 2000 rpm. 

**Figure 5:**
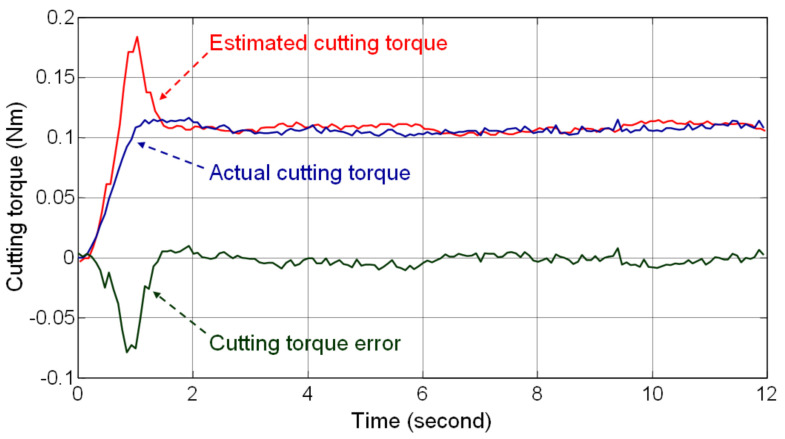
Estimated and actual cutting torque in the DOB tests.

Figure 5 shows the testing results and demonstrates that the bandwidth of the low-pass filter $Q\left(s\right)$ is closely related to the transient response of the estimated cutting torque. Filter $Q\left(s\right)$ was designed with high bandwidth to rapidly estimate the cutting torque; therefore, a large overshoot occurred in the estimated cutting torque when the thread milling blade came in contact with the workpiece. However, the error in the estimated cutting torque reduced rapidly when the milling reached a steady-state condition. The average value of the steady-state error in the cutting torque was −0.0016 Nm, which is 1.45% of the average value of the actual steady-state cutting torque. The root mean square value of the steady-state error in the cutting torque was 0.0045 Nm, which is 4.19% of the average value of the actual steady-state cutting torque. Therefore, the DOB tests evaluate the steady-state accuracy of the estimated cutting torque and validate the feasibility of using the DOB for estimating the cutting torque in thread milling. 

### 4.3. Thread Milling Experiments

Thread milling experiments were performed on the workpiece with stacked materials of AL6061 and AL7075 to validate the feasibility of the SSM control designed in this study. The workpiece with stacked materials was prepared by stacking the AL6061 (upper part) and AL7075 (lower part) materials. Thread milling of AL6061 (upper part) was the first step of the experiment, followed by the milling of AL7075 (lower part). The cutting feedrate used in the experiment was 300 mm/min and the depth of cut was 1.5 mm. The preset torque command of the SSM control was 0.07 Nm.

**Figure 6:**
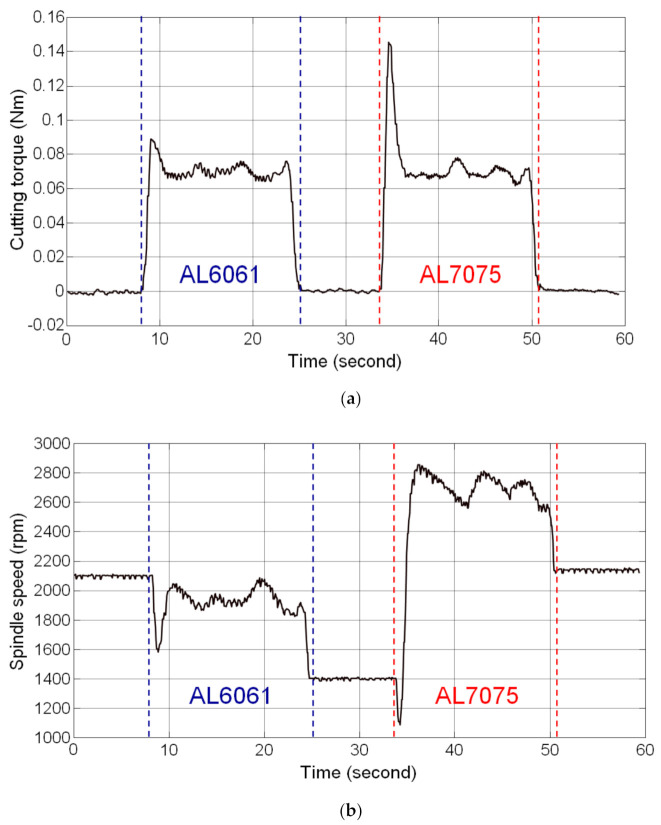
Results of thread milling experiments on the workpiece with stacked materials (SSM control). (**a**) Estimated cutting torque; (**b**) Actual spindle speed.

The experimental results are shown in Figure 6. In the AL6061 thread milling stage, an overshoot occurred in the estimated cutting torque because the thread milling blade came in contact with the workpiece. The estimated cutting torque was 0.0890 Nm. However, the magnitude of the cutting torque approached the preset torque command when the thread milling was in a steady-state condition. The average value of the estimated cutting torque in the steady-state condition was 0.0694 Nm and the steady-state error was 6.1922 × 10^−4^ Nm (percentage error: 0.88%). The cutting torque fluctuated depending on the actual cutting conditions and the root mean square value of estimated cutting torque fluctuation was 0.0028 Nm. In the AL7075 thread milling stage, a larger overshoot occurred in the estimated cutting torque (0.1453 Nm) when the thread milling blade came in contact with the workpiece because AL7075 is harder than AL6061. In steady-state thread milling, the average value of the estimated cutting torque was 0.0691 Nm, the steady-state error was 9.2477 × 10^−4^ Nm (percentage error: 1.32%), and the root mean square value of the estimated cutting torque fluctuation was 0.0032 Nm. The estimated cutting torque shown in Figure 6a shows that the SSM control enables the cutting torque to reach the preset torque command when applied to thread milling of different materials; moreover, during thread milling of different materials, the spindle speed was modulated automatically on the basis of the estimated cutting torque to maintain the cutting torque at the preset torque command, as shown in Figure 6b. Therefore, the results of the thread milling experiments on the workpiece with stacked materials validated the feasibility of the SSM control designed in this study. 

**Figure 7:**
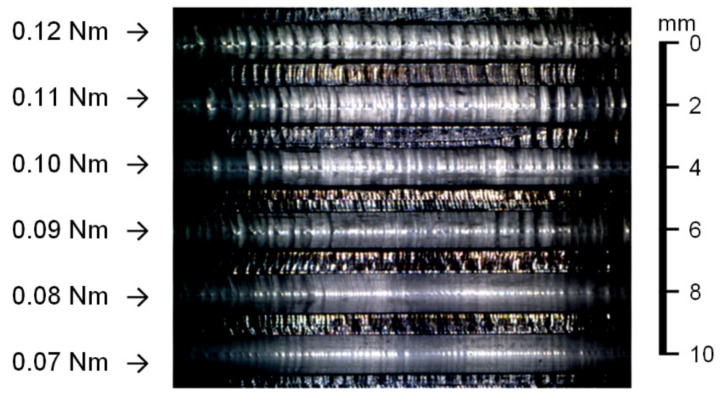
Results of the thread milling experiments on the AL6061 workpiece (preset torque command changed in the range of 0.07–0.12 Nm).

**Figure 8:**
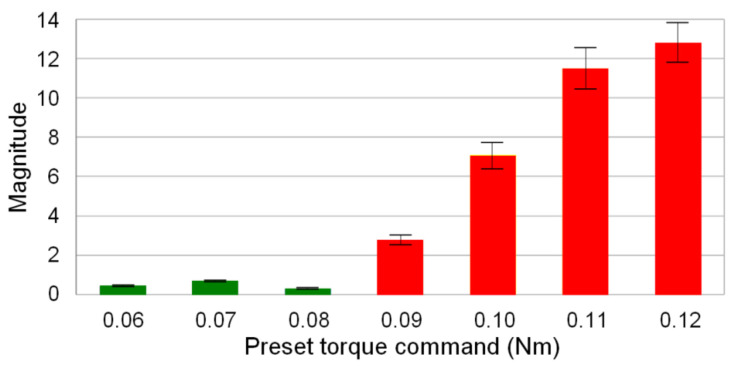
Variation in the magnitude of the main frequency of cutting vibrations.

**Figure 9:**
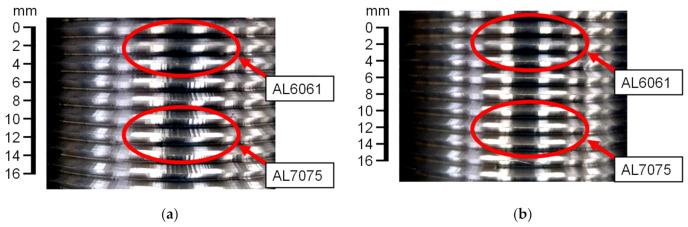
Results of the thread milling experiments on the workpiece with stacked materials (CVS control). (**a**) Without CVS control; (**b**) with CVS control.

The thread milling experiments conducted on the AL6061 workpiece indicate that cutting vibrations occur if the preset torque command of the SSM control is greater than 0.08 Nm. As shown in Figure 7, cutting marks were observed in this condition. The main frequency of cutting vibrations was obtained by FFT of the estimated cutting torque signal. Figure 8 shows that the magnitude corresponding to the main frequency of the cutting vibrations increases as the preset torque command is increased. Therefore, thread milling experiments were performed to validate the feasibility of the CVS control for suppressing the cutting vibrations during thread milling. The cutting feedrate was 300 mm/min and the depth of cut was 1.5 mm. Figure 9a shows the experimental results obtained without CVS control (preset torque command was 0.05 Nm). No cutting vibrations occurred during thread milling of the AL6061 workpiece. However, cutting vibrations occurred during thread milling of the AL7075 workpiece owing to the large cutting torque. Figure 9b shows the experimental results obtained using the CVS control. During thread milling of the AL6061 workpiece using the CVS control, the cutting torque did not cause any cutting vibration because it was maintained at the preset torque command. When the CVS control detected cutting vibrations, it automatically adjusted the torque command to suppress the vibrations. The threads obtained using the CVS control exhibited smoother surfaces compared with the threads obtained without CVS control. When the CVS control was used during thread milling, the manufactured threads of the workpiece with stacked materials did not have any cutting marks. Therefore, the thread milling experiments validated that the integrated design of SSM and CVS controls can effectively suppress cutting vibrations during thread milling.

## 5. Conclusions

This study developed an integrated design of SSM and CVS controls using a DOB, to automatically modulate the spindle motion characteristics for controlling the cutting torque while avoiding the cutting vibration during thread milling. The proposed SSM control utilized a DOB to estimate the cutting torque on the spindle during thread milling, and the estimated cutting torque was used as the feedback signal for the SSM control to modulate the spindle speed so that the cutting torque during thread milling was maintained at the preset torque command. The CVS control designed on the basis of the SSM control utilized FFT to analyze the cutting torque signal estimated by the DOB to detect the occurrence of cutting vibrations, and then adjusted the torque command of the SSM control to suppress the cutting vibrations when cutting vibrations occur during thread milling. 

Several experiments were performed on a CNC milling machine to validate the feasibility and performance of the proposed integrated design. The experimental results indicated that the average values of the error in the cutting torque in the steady-state condition were less than 2% when the SSM control was performed on the thread milling with different materials. The experimental results on the CVS control exhibited no apparent cutting marks on the manufactured thread surfaces, even for a workpiece stacked with different materials. To efficiently manufacture threads, machining conditions were usually set to provide a large cutting torque on the spindle, which caused cutting vibrations and degraded the thread quality (e.g., apparent cutting marks on the surface of the manufactured threads). Therefore, it was difficult to achieve high thread manufacturing efficiency and superior thread quality in thread milling, owing to the diverse machining conditions and workpiece materials. The integrated design developed in this study can thus be applied to thread milling processes to improve the thread manufacturing efficiency and thread quality, and to mitigate the cost and installation problems prevalent in the existing sensor-based methods. Furthermore, considering the development of monitoring-simulation systems for improving dimensional and surface accuracy of manufactured parts [41,42] the cutting torque estimation and analyzing methods developed in this study for thread milling can be applied to monitoring-simulation systems to further improve the thread manufacturing efficiency and quality.

## Data Availability

Not applicable.

## Appendix A

Referring to Equations (7) and (12) in Section 3.2, Equation (A1) can be obtained.

(A1) $$\begin{array}{l} \left[\begin{array}{cc} K_{t}^{-1}K_{m}+G & Q^{-1}\left(1-Q\right)-\left(K_{t}^{-1}{\hat{B}}_{m}+G\right)\;A\\ -\left(G_{m}-K_{m}\right) & K_{t}Q^{-1} \end{array}\right]\;\left[\begin{array}{c} \omega_{m}\\ I_{m}^{DOB} \end{array}\right]\\ =\left[\begin{array}{c} K_{t}^{-1}{\hat{\tau }}_{m}^{sc}+\left(K_{t}^{-1}{\hat{B}}_{m}+G\right)\;\left(\omega_{m}^{c}-AI_{m}^{r}\right)\\ \tau_{m}^{d} \end{array}\right] \end{array}$$

Equations (A2) and (A3) can be obtained from Equation (A1).

(A2) $$-\left(G_{m}-K_{m}\right)\;\omega_{m}+K_{t}Q^{-1}I_{m}^{DOB}=\tau_{m}^{d}$$

(A3) $$\begin{array}{l} \left(K_{t}^{-1}K_{m}+G\right)\;\omega_{m}+\left[Q^{-1}\left(1-Q\right)-\left(K_{t}^{-1}{\hat{B}}_{m}+G\right)\;A\right]\;I_{m}^{DOB}\\ =K_{t}^{-1}{\hat{\tau }}_{m}^{sc}+\left(K_{t}^{-1}{\hat{B}}_{m}+G\right)\;\left(\omega_{m}^{c}-AI_{m}^{r}\right) \end{array}$$

Furthermore, Equation (A4) can be deduced from Equations (A2) and (A5) can be deduced from Equations (A3) and (A4).

(A4) $$I_{m}^{DOB}=K_{t}^{-1}Q\tau_{m}^{d}+K_{t}^{-1}Q\;\left(G_{m}-K_{m}\right)\;\omega_{m}$$

(A5) $$\begin{array}{l} \left(K_{t}^{-1}K_{m}+G\right)\;\omega_{m}\\ +\left[Q^{-1}\left(1-Q\right)-\left(K_{t}^{-1}{\hat{B}}_{m}+G\right)\;A\right]\;\left[K_{t}^{-1}Q\tau_{m}^{d}+K_{t}^{-1}Q\;\left(G_{m}-K_{m}\right)\;\omega_{m}\right]\\ =K_{t}^{-1}{\hat{\tau }}_{m}^{sc}+\left(K_{t}^{-1}{\hat{B}}_{m}+G\right)\;\left(\omega_{m}^{c}-AI_{m}^{r}\right). \end{array}$$

Based on Equation (A6) given below,

(A6) $$\begin{array}{l} \left[Q^{-1}\left(1-Q\right)-\left(K_{t}^{-1}{\hat{B}}_{m}+G\right)\;A\right]\;\left[K_{t}^{-1}Q\tau_{m}^{d}+K_{t}^{-1}Q\left(G_{m}-K_{m}\right)\;\omega_{m}\right]\\ =\left[K_{t}^{-1}\left(1-Q\right)-\left(K_{t}^{-1}{\hat{B}}_{m}+G\right)\;AK_{t}^{-1}Q\right]\tau_{m}^{d}\\ +\left[K_{t}^{-1}\left(1-Q\right)\left(G_{m}-K_{m}\right)-\left(K_{t}^{-1}{\hat{B}}_{m}+G\right)AK_{t}^{-1}Q\;\left(G_{m}-K_{m}\right)\right]\;\omega_{m} \end{array}$$

Equation (A5) can be rewritten as Equation (A7).

(A7) $$\begin{array}{l} \left(K_{m}+K_{t}G\right)\;\omega_{m}\\ +\left[\left(1-Q\right)-\left(K_{t}^{-1}{\hat{B}}_{m}+G\right)AQ\right]\;\tau_{m}^{d}\\ +\left[\left(1-Q\right)\left(G_{m}-K_{m}\right)-\left(K_{t}^{-1}{\hat{B}}_{m}+G\right)AQ\left(G_{m}-K_{m}\right)\right]\;\omega_{m}\\ ={\hat{\tau }}_{m}^{sc}+K_{t}\left(K_{t}^{-1}{\hat{B}}_{m}+G\right)\;\left(\omega_{m}^{c}-AI_{m}^{r}\right) \end{array}$$

Because

(A8) $$\begin{array}{l} \left[\left(1-Q\right)-\left(K_{t}^{-1}{\hat{B}}_{m}+G\right)AQ\right]\;\tau_{m}^{d}\\ =\tau_{m}^{d}-\left[1+\left(K_{t}^{-1}{\hat{B}}_{m}+G\right)A\right]\;Q\tau_{m}^{d} \end{array}$$

and

(A9) $$\begin{array}{l} \left[\left(1-Q\right)\left(G_{m}-K_{m}\right)-\left(K_{t}^{-1}{\hat{B}}_{m}+G\right)AQ\left(G_{m}-K_{m}\right)\right]\;\omega_{m}\\ =\left(G_{m}-K_{m}\right)\;\omega_{m}\\ -\left[1+\left(K_{t}^{-1}{\hat{B}}_{m}+G\right)A\right]\;Q\left(G_{m}-K_{m}\right)\;\omega_{m} \end{array}$$

Equation (A7) can be rewritten as Equations (A10) and (A11).

(A10) $$\begin{array}{l} \left(K_{m}+K_{t}G\right)\;\omega_{m}+\tau_{m}^{d}-Q\tau_{m}^{d}-\left(K_{t}^{-1}{\hat{B}}_{m}+G\right)\;AQ\tau_{m}^{d}\\ +\left(G_{m}-K_{m}\right)\;\omega_{m}-Q\left(G_{m}-K_{m}\right)\;\omega_{m}\\ -\left(K_{t}^{-1}{\hat{B}}_{m}+G\right)\;AQ\left(G_{m}-K_{m}\right)\;\omega_{m}\\ ={\hat{\tau }}_{m}^{sc}+K_{t}\left(K_{t}^{-1}{\hat{B}}_{m}+G\right)\;\left(\omega_{m}^{c}-AI_{m}^{r}\right) \end{array}$$

(A11) $$\begin{array}{l} \left(K_{m}+K_{t}G\right)\;\omega_{m}\\ +\left[1-Q-\left({\hat{B}}_{m}+K_{t}G\right)\;AK_{t}^{-1}Q\right]\;\left(G_{m}-K_{m}\right)\;\omega_{m}\\ =\left({\hat{\tau }}_{m}^{sc}-\tau_{m}^{sc}\right)-\tau_{m}^{e}+Q\tau_{m}^{d}\\ +\left({\hat{B}}_{m}+K_{t}G\right)\;\left[\left(\omega_{m}^{c}-AI_{m}^{r}\right)+AK_{t}^{-1}Q\tau_{m}^{d}\right] \end{array}$$

Here, the external disturbance torque $\tau_{m}^{d}$ comprises the disturbance torque $\tau_{m}^{sc}$ and actual cutting torque $\tau_{m}^{e}$, that is, $\tau_{m}^{d}=\tau_{m}^{sc}+\tau_{m}^{e}$. Because of Equation (A12),

(A12) $$\begin{array}{l} \left({\hat{\tau }}_{m}^{sc}-\tau_{m}^{sc}\right)-\tau_{m}^{e}+Q\tau_{m}^{d}\\ +\left({\hat{B}}_{m}+K_{t}G\right)\;\left[\left(\omega_{m}^{c}-AI_{m}^{r}\right)+AK_{t}^{-1}Q\tau_{m}^{d}\right]\\ =\left({\hat{\tau }}_{m}^{sc}-\tau_{m}^{sc}\right)-\left(1-Q\right)\;\tau_{m}^{e}\\ +\left({\hat{B}}_{m}+K_{t}G\right)\;\left[\left(\omega_{m}^{c}-AI_{m}^{r}\right)+\left(AK_{t}^{-1}+{\left({\hat{B}}_{m}+K_{t}G\right)}^{-1}\right)Q\tau_{m}^{sc}+AK_{t}^{-1}Q\tau_{m}^{e}\right] \end{array}$$

Equation (A11) can be rewritten as Equation (A13).

(A13) $$\begin{array}{l} \left(K_{m}+K_{t}G\right)\;\omega_{m}\\ +\left[1-Q-\left({\hat{B}}_{m}+K_{t}G\right)\;AK_{t}^{-1}Q\right]\;\left(G_{m}-K_{m}\right)\;\omega_{m}\\ =\left({\hat{\tau }}_{m}^{sc}-\tau_{m}^{sc}\right)-\left(1-Q\right)\;\tau_{m}^{e}\\ +\left({\hat{B}}_{m}+K_{t}G\right)\;\left[\left(\omega_{m}^{c}-AI_{m}^{r}\right)+\left(AK_{t}^{-1}+{\left({\hat{B}}_{m}+K_{t}G\right)}^{-1}\right)Q\tau_{m}^{sc}+AK_{t}^{-1}Q\tau_{m}^{e}\right] \end{array}$$

The speed command compensation $\omega_{m}^{c}$ is expressed as Equation (A14).

(A14) $$\omega_{m}^{c}=\omega_{m}^{ref}-{\left({\hat{B}}_{m}+K_{t}G\right)}^{-1}Q\;{\hat{\tau }}_{m}^{sc}-AK_{t}^{-1}Q\;\left({\hat{\tau }}_{m}^{sc}+{\hat{\tau }}_{m}^{e}\right)$$

Equation (A13) can then be rewritten as Equation (A15).

(A15) $$\begin{array}{l} \left(K_{m}+K_{t}G\right)\;\omega_{m}\\ +\left[1-Q-\left({\hat{B}}_{m}+K_{t}G\right)\;AK_{t}^{-1}Q\right]\;\left(G_{m}-K_{m}\right)\;\omega_{m}\\ =\left({\hat{\tau }}_{m}^{sc}-\tau_{m}^{sc}\right)-\left(1-Q\right)\;\tau_{m}^{e}\\ +\left({\hat{B}}_{m}+K_{t}G\right)\;\left[\left(\omega_{m}^{\mathrm{ref}}-AI_{m}^{r}\right)-\left(AK_{t}^{-1}+{\left({\hat{B}}_{m}+K_{t}G\right)}^{-1}\right)\;Q\;\left({\hat{\tau }}_{m}^{sc}-\tau_{m}^{sc}\right)-AK_{t}^{-1}Q\;\left({\hat{\tau }}_{m}^{e}-\tau_{m}^{e}\right)\right] \end{array}$$
